# Supplementary material for: Sorting at embryonic boundaries requires high heterotypic interfacial tension
Source: Nat Commun. 2017 Jul 31;8:157. doi: 10.1038/s41467-017-00146-x (PMC5537356; doi:10.1038/s41467-017-00146-x)
Supplement: Supplementary file 2 — Supplementary Software 1 [file 41467_2017_146_MOESM2_ESM.zip › PottsModel/SrcPottsModel/doc/model/CellSet.html]

CellSet


JavaScript is disabled on your browser.


Skip navigation links


- Overview
- Package
- Class
- Use
- Tree
- Deprecated
- Index
- Help

- Prev Class
- Next Class

- Frames
- No Frames

- All Classes

- Summary:
- Nested |
- Field |
- Constr |
- Method

- Detail:
- Field |
- Constr |
- Method


model

## Class CellSet

- java.lang.Object
- - model.CellSet

- All Implemented Interfaces:
  :   java.lang.Iterable<Cell>

  ---

    

  ```
  public class CellSet
  extends java.lang.Object
  implements java.lang.Iterable<Cell>
  ```

- - ### Constructor Summary

    Constructors

    Constructor and Description

    `CellSet()`
  - ### Method Summary

    All Methods Static Methods Instance Methods Concrete Methods

    | Modifier and Type | Method and Description |
    | --- | --- |
    | `void` | `addCell(Cell pCell)` Adds a cell to the CellSet. |
    | `boolean` | `contains(Cell pCell)` Function used when reading in the grid. |
    | `boolean` | `contains(int pCellID)` Whether the cell set contains a cell with given ID. |
    | `double` | `getAreaEnergy()` Gets the cumulative area energy of all cells in the system. |
    | `Cell` | `getCell(int pCellID)` |
    | `static double` | `getLocalAreaEnergy(Cell pCell1, Cell pCell2)` Gets the area energy of the two cells passed as parameters. |
    | `static double` | `getLocalPerimeterEnergy(Cell pCell1, Cell pCell2)` Gets the perimeter energy of the two cells passed as parameters. |
    | `double` | `getPerimeterEnergy()` Gets the cumulative perimeter energy of all cells in the system. |
    | `java.util.Iterator<Cell>` | `iterator()` |
    | `void` | `removeCell(Cell pCell)` |
    | `void` | `removeCell(int pCellId)` |
    | `int` | `size()` |

    - ### Methods inherited from class java.lang.Object

      `equals, getClass, hashCode, notify, notifyAll, toString, wait, wait, wait`
    - ### Methods inherited from interface java.lang.Iterable

      `forEach, spliterator`

- - ### Constructor Detail


    - #### CellSet

      ```
      public CellSet()
      ```
  - ### Method Detail


    - #### addCell

      ```
      public void addCell(Cell pCell)
      ```

      Adds a cell to the CellSet. No two cells should be allowed to share the same id.

      Parameters:
      :   `pCell` - The Cell to add to the CellSet.


    - #### removeCell

      ```
      public void removeCell(Cell pCell)
      ```


    - #### removeCell

      ```
      public void removeCell(int pCellId)
      ```


    - #### getPerimeterEnergy

      ```
      public double getPerimeterEnergy()
      ```

      Gets the cumulative perimeter energy of all cells in the system.

      Returns:
      :   The sum of the perimeter energies of individual cells.
          TODO: how about perimeter energy of the cell cluster i.e. do not count boundary pixels within cluster?


    - #### getAreaEnergy

      ```
      public double getAreaEnergy()
      ```

      Gets the cumulative area energy of all cells in the system.

      Returns:
      :   The sum of the area energies of individual cells.


    - #### getLocalAreaEnergy

      ```
      public static double getLocalAreaEnergy(Cell pCell1,
                                              Cell pCell2)
      ```

      Gets the area energy of the two cells passed as parameters.

      Returns:
      :   The sum of the two area energies calculated.


    - #### getLocalPerimeterEnergy

      ```
      public static double getLocalPerimeterEnergy(Cell pCell1,
                                                   Cell pCell2)
      ```

      Gets the perimeter energy of the two cells passed as parameters.

      Returns:
      :   The sum of the two perimeter energies calculated.


    - #### contains

      ```
      public boolean contains(Cell pCell)
      ```

      Function used when reading in the grid. Checks whether the given
      cell ID has been read in already (so we are adding to this cell)
      or whether we need to create a new cell with this ID.

      Parameters:
      :   `cellID` - The cell id we are checking.

      Returns:
      :   whether that cell has already begun to be read in.


    - #### contains

      ```
      public boolean contains(int pCellID)
      ```

      Whether the cell set contains a cell with given ID.

      Parameters:
      :   `pCellID` -

      Returns:
      :   true if cell set contains cell with the given ID


    - #### getCell

      ```
      public Cell getCell(int pCellID)
      ```


    - #### iterator

      ```
      public java.util.Iterator<Cell> iterator()
      ```

      Specified by:
      :   `iterator` in interface `java.lang.Iterable<Cell>`


    - #### size

      ```
      public int size()
      ```


Skip navigation links


- Overview
- Package
- Class
- Use
- Tree
- Deprecated
- Index
- Help

- Prev Class
- Next Class

- Frames
- No Frames

- All Classes

- Summary:
- Nested |
- Field |
- Constr |
- Method

- Detail:
- Field |
- Constr |
- Method
